# Book Review: The Spontaneous Brain: From the Mind-Body to the World-Brain Problem

**DOI:** 10.3389/fpsyg.2021.644237

**Published:** 2021-02-22

**Authors:** Da Dong, Jing Zhang

**Affiliations:** ^1^Department of Philosophy, Center for the Study of Language and Cognition, Zhejiang University, Hangzhou, China; ^2^Institute of Philosophy, Hangzhou Dianzi University, Hangzhou, China

**Keywords:** spontaneous activity, spatiotemporal neuroscience, mind-body problem, world-brain problem, Copernican revolution in neuroscience and philosophy

The problem of consciousness is a controversial topic lacking a broadly accepted definition in contemporary discussions. Views about what the mind-body problem is and how it could be properly resolved, diverge widely. The neuroscientist Georg Northoff asserts that, without having an idea of the *world*— “what the brain's inside of,” (borrow the term from Mace, 1977) the problem is a hopeless task. In *The Spontaneous Brain: From the Mind-Body to the World-Brain Problem* (Northoff, 2018), Northoff argues that a promising approach to tackle the existence and reality of mental features needs to shift the focus from the mind-body relationship to world-brain relationship.

The book focuses on how and why the brain and its spontaneous activity (or resting-state activity) could be a “game-changer” (p. 435) on the issue of mental features. In short, Northoff attempts to answer the following two questions: (1) why is the mind-body problem not the right way to inquire about mental features? (2) why is the world-brain problem could be a preferable alternative? Meanwhile, he offers a wealth of empirical evidence on the brain's spontaneous activity (rather than stimulus-induced activity) which stands as the foundation of consciousness.

A helpful overview of the book: the book divides into five parts. In the “Introduction,” Northoff points out that he aims to “change and shift our focus from brain and mind to world-brain relation as a necessary condition of mental features, specifically consciousness.” (p. xi) Northoff does not intend to find an answer to the question of mind-brain relations; instead, he questions the question itself (p. xii). Then he turns to explore the ontological relations of the brain and the world, thus the dubbed “world-brain problem.” For this purpose, Northoff argues separately from three different grounds (corresponds to the three parts of the book): empirical (Part I and Part II), ontological (Part III), and epistemic-methodological (Part IV).

Part I (“Models of Brain,” Chapter 1–3) tries to sketch a new model of the brain. It starts from the debates between two kinds of brain models, what Northoff terms as passive and active (Chapter 1). For Northoff, an appropriate model of brain would be able to explain integrations and balances (or tradeoffs) between stimulus-induced activity and spontaneous activity. The next two chapters further demonstrate that an essential character of a brain model should be interactive rather than parallel between spontaneous and task activity. Thus, Northoff argues that the brain (model) should be treated as an open and world-evidencing system rather than a closed (or secluded) and self-evidencing system.

Part II (“Models of Consciousness,” Chapter 4–8) goes on to reveal how the spectrum model of brain (Chapter 4 as based on Chapter 1), the interaction model of brain (Chapter 5 as based on Chapter 2), and the prediction model of brain (Chapter 6 as based on Chapter 3) relate to consciousness, respectively. Chapter 7 offers a spatiotemporal theory of consciousness, which focuses first and foremost on the spatio-temporal features of the brain. This theory is different from content-based, cognitive, and informational theory of consciousness, which includes four mechanisms: spatiotemporal nestedness, expansion, globalization, and alignment. Finally, Chapter 8 emphasizes the central role of the brain's alignment to both the body and the world.

Part III (“World-Brain Problem,” Chapter 9–11) centers on the ontological level of the project. In this part, Northoff attempts to show that the brain and its spontaneous activity are also an ontological game-changer. Ontologically, the brain which should be understood in terms of its relation to the world. Part III elaborates how a version of ontic structural realism could help conceive those relations in Northoff's enterprise.

In Part IV (“Copernican Revolution,” Chapter 12–14), Northoff argues that the transition from mind-body problem to world-brain problem is essential for us to understand various mental features, how our brain becomes part of the world and then how we become part of the world. Thus, analogically, Northoff concludes that his project requires “a Copernican revolution in neuroscience and philosophy.” (p. xii) As he says, “we need to replace our current vantage point from within mind (or within brain) to a vantage point from beyond brain,” (pp. xxii–xxiii) thus drawing on a similar historical study of the Copernican revolution in astronomy (p. xxvi).

The key concept of “world-brain relation” might be involved in at least three senses: mereologically, a brain is one proper part (in a similar way of proper set in set theory) of the world; geometrically, a brain is one fractal of the world; dynamically, a self-organizing brain (and its spontaneous activity) is a natural continuity of the dynamical world. In “Conclusion,” Northoff asserts, the triad of (mereological/fractal/dynamical) brain as well as its self-organizing spontaneous activity are a game-changer in neuroscience and philosophy. Again, the core of his project is to construct a spatiotemporal model of brain and consciousness “with a central role for world-brain relation.” (p. 435).

As discussed above, it is almost evident that Northoff is not an eliminativist; he is a dynamicist on the all-out ontological continuity between brain and “what the brain's inside of.” In another sense, Northoff also embraces a theory of ontic structural realism; he states that structures and relations are the ultimate units of reality (both for brain and world) (p. xviii). Even on the replacement of the mind-brain problem with the world-brain problem, Northoff argues that in his conceptual framework, the conventional uses of terminologies of “mind” are less fundamental and thus replaceable. Anyhow, this replacement is a little too weird for most people.

In conclusion, although Northoff's work has received less attention so far, his approach deserves more serious consideration from psychologists, neuroscientists, and philosophers. Besides, we recommend newly published journal articles by Northoff et al. (2020a,b), which represent his latest theoretical developments about shared features of brain and mind, that is their “common currency.”

## Author Contributions

DD and JZ wrote the manuscript, with larger contributions by DD. JZ then provided edits and suggestions for revision. All authors contributed to the article and approved the submitted version.

## Conflict of Interest

The authors declare that the research was conducted in the absence of any commercial or financial relationships that could be construed as a potential conflict of interest.
